# *Renibacterium salmoninarum*—The Causative Agent of Bacterial Kidney Disease in Salmonid Fish

**DOI:** 10.3390/pathogens9100845

**Published:** 2020-10-15

**Authors:** Mohammad Reza Delghandi, Mansour El-Matbouli, Simon Menanteau-Ledouble

**Affiliations:** Clinical Division of Fish Medicine, University of Veterinary Medicine, Veterinärplatz 1, 1210 Vienna, Austria; Mohammad.delghandi@vetmeduni.ac.at (M.R.D.); Mansour.el-matbouli@vetmeduni.ac.at (M.E.-M.)

**Keywords:** facultative intracellular, vertical transmission, asymptomatic carriers, chronic infections, granuloma, p57

## Abstract

*Renibacterium salmoninarum* is one of the oldest known bacterial pathogens of fish. This Gram-positive bacterium is the causative agent of bacterial kidney disease, a chronic infection that is mostly known to infect salmonid fish at low temperatures. Externally, infected fish can display exophthalmia as well as blebs on the skin and ulcerations alongside haemorrhages at the base of the fins and alongside the lateral line. Internally, the kidney, heart, spleen and liver can show signs of swelling. Granulomas can be seen on various internal organs, as can haemorrhages, and the organs can be covered with a false membrane. Ascites can also accumulate in the abdominal cavity. The bacterium is generally cultivated on specialized media such as kidney disease medium-1 (KDM-1), KDM-2 and selective kidney disease medium (SKDM), and a diagnostic is performed using molecular tools such as PCRs or real-time quantitative PCRs (RT-qPCRs). Several virulence mechanisms have been identified in *R. salmoninarum*, in particular the protein *p57* that is known to play a role in both agglutination and immunosuppression of the host’s defense mechanisms. Control of the disease is difficult; the presence of asymptomatic carriers complicates the eradication of the disease, as does the ability of the bacterium to gain entrance inside the eggs. Bacterin-killed vaccines have proven to be of doubtful efficacy in controlling the disease, and even more recent application of a virulent environmental relative of *R. salmoninarum* is of limited efficacy. Treatment by antibiotics such as erythromycin, azithromycin and enrofloxacin can be effective but it is slow and requires prolonged treatment. Moreover, antibiotic-resistant strains have been reported. Despite being known for a long time, there is still much to be discovered about *R. salmoninarum*, notably regarding its virulence mechanisms and its vaccine potential. Consequently, these gaps in knowledge continue to hinder control of this bacterial disease in aquaculture settings.

## 1. Introduction 

*Renibacterium salmoninarum* is the causative agent of bacterial kidney disease (BKD), an important disease, particularly in salmonid fish worldwide [1,2]. The causative agent can be transmitted both vertically and horizontally [3,4,5]. Wild and cultured salmonids can be affected by *R. salmoninarum* at all stages of life and it has a serious impact on the salmonid industry [6,7,8]. *R. salmoninarum* has been associated with mortalities as high as 80% in Pacific salmon (*Oncorhynchus* spp.) and 40% in Atlantic salmon (*Salmo salar*) [9]. In some extreme cases, daily mortality rates as high as 5% have been reported [10,11], mostly in juvenile salmons 6 to 12 months old and pre-spawning adults [8,12]. Several risk factors can influence bacterial proliferation and the development of the disease, including ecological features, geographical region, ecosystem, food condition, size and age of fish [13]. For example, the occurrence of the disease is significantly higher in the summer season [4]. Moreover, the time of the year in which fish were transferred out of the hatchery also influence their likelihood of developing a *R. salmoninarum* infection with fish stocked in the spring and summer being significantly more likely to develop BKD. This could be possibly explained as the fish being younger [4] and more susceptible at the peak period in which the bacterium was more active. 

Prevention and control of *R. salmoninarum* is difficult as no vaccines are reliably efficacious. Moreover, antibiotic resistance is another issue. Prevention can be performed through good husbandry practice [14,15]. The severity of infections can range from subclinical to acute [4] and *R. salmoninarum* has been associated with subclinical infectious in salmonid populations but the bacterium can be detected in the kidney and surface mucus at this stage [16].

Despite being an old foe for salmon and trout farmers, much remains to be elucidated about *R. salmoninarum*. This is partly due because of the disease fastidiousness and slow-growing nature that makes research difficult. Moreover, other pathogens cause more acute and “spectacular” losses, which likely contributed to them generating more interest. Nonetheless, *R. salmoninarum* remains an important fish pathogen and research efforts need to be continued regarding this vexing disease. 

## 2. Search Strategy

This literature review is based on PubMed (http://www.ncbi.nlm.nih.gov/pubmed), Google scholar (http://scholar.google.com) as well as Scopus^®^ (http://www.scopus.com) using thematic key words and other terms related to this review (e.g., bacterial kidney disease, virulence factor, transmission, clinical signs and treatment). The articles were investigated and evaluated in detail. Furthermore, we made use of several reference books on the subject of fish. 

## 3. Historical Background and Classification

BKD was first reported in wild Atlantic salmon in the Rivers Dee and Spey in Scotland in the 1930s. In 1935, a similar syndrome was identified in North America in brook trout (*Salvelinus fontinalis*), brown trout (*Salmo trutta*) and rainbow trout (*Oncorhynchus mykiss*) [17,18,19,20]. The same year, Belding and Merrlli reported a Gram-positive bacterium isolated from necrotic kidney lesions consistent with *R. salmoninarum* in salmonids in the United States, although these authors were not able to culture the bacterial agent [17].

*R. salmoninarum* is a Gram-positive, non-motile, non-capsulated, non-endospores forming, aerobic, non-acid fast small diplobacillus (0.3 to 1.0 by 1.0 to 1.5 µm) [21]. The bacterium was initially considered to belong to the *Corynebacterium* genus. However, in contrast with *Corynebacterium*, the cell wall of *R. salmoninarum* does not contain mycolic acids. Moreover, comparison between *R. salmoninarum* and *Listeria monocytogenes* showed that the cell wall of *Listeria monocytogenes* contains mesodiaminopimelic acid, aspartic acid, and leucine, while the *R. salmoninarum* cell lacks these peptidoglycans [21,22,23]. It was recognized on the basis of its major cell wall’s amino acids, in particular, alanine, glutamic acid, lysine and glycine that the organism belongs to its own genus, where it is the sole species.

Infections by *R. salmoninarum* have been described under several names over the year, partly reflecting its changing position in nomenclature; these include corynebacterial disease, Dee disease, kidney disease, white boil disease and salmonid kidney disease [22,24]. However, bacterial kidney disease is the term most commonly used today.

The genome of *R. salmoninarum* strain ATCC 33209^T^ is 3.15 Mbp in length and most closely related to that of the environmental bacterial genus *Arthrobacter*, although the genomes of *Arthrobacter* spp. strain FB24 and *Arthrobacter aurescens* TC1 genomes are 1.9 Mb bigger and have a higher G+C content. Both microorganisms belong to the *Micrococcaceae* family [25]. According to 16S rRNA sequence, *R. salmoninarum* has also been classified in the *Micrococcus-Arthrobacter* subdivision of the actinomycetes [26].

Comparison between *R. salmoninarum* isolates from Atlantic salmon and rainbow trout with strains related to Dee disease in Scotland using variable number tandem repeat analysis suggests that *R. salmoninarum* isolated in European aquaculture differ from wild strains. Matejusova et al. [19] have reported that *R. salmoninarum* haplotypes comprised two groups. The first one was isolated from Scotland and Norway aquacultures and included isolates related to the strain ATCC 33209^T^, originally isolated from Chinook salmon (*Oncorhynchus tshawytscha*) in North America. The other group includes NCIBM 1114 and NCIBM 1116 strains that were also isolated from wild Atlantic salmon and related to Dee disease [19]. Similarly, investigations of the *R. salmoninarum* phylogenies were performed on isolates from several salmonid species using single-nucleotide polymorphism (SNP) and showed that this bacterium includes two lineages. Lineage 1 divided into two sub lineages, 1b originating from North America and 1a originating from Norway, Canada, and the UK, and further divided into six clusters (UK1, UK2, UK3NA1, NA2 and UK/NOR1). Meanwhile, Lineage 2 is mostly confined to waters in continental Europe [27]. Brynildsrud et al. (2016) investigated the role of copy number variation (CNV) in virulence factor *msa* and *p22* and reported that CNVs were affected by geographical origin and phylogenetic relationship. The authors found that *msa* gene exists in the form of two to five copies and the *p22* in the form of one to five copies. In this study, CNVs were observed in different isolates, such as 5006, 5223, 05372K, BQ96_91, BPS91, Carson5b, Cow-Chs-94, GR5, RS2, RS6 and RS10. They discovered two *msa* duplication types (type I and type II), while no *p12* were observed in type II. CNV has not been observed in Lineage 2 and on the other hand CNV existed in some strains isolated from the Pacific Northwest, Eastern North America and Norway. However, there was no correlation between *msa-p22* duplication with host species, years of isolation and fresh or salt water habitat [28].

Similarly, Bayliss et al. [1] applied whole genome sequencing (WGS) to construct a phylogenetic tree for 42 *R. salmoninarum* isolates identified in Chile between 2012 and 2016. These authors were able to identify four groups corresponding to the multiple introductions of this bacterium from different countries over the course of the years. This study demonstrated the importance of fish transfer in the international spread of the bacterium [1].

## 4. Distribution of the Disease

### 4.1. Geographical and Temporal Distribution

BKD has been reported in salmonids stock culture from Japan, South America, North America and Europe. This disease has not been reported in Ireland, Australia, New Zealand, which is likely due to it being a freshwater pathogen. More unexpectedly, the former Soviet Union has also not been associated with outbreaks of the disease [6,24,29].

*R. salmoninarum* is a cold-water pathogen and it is most active at a temperature of 15 °C or below. When comparing the severity of the disease at 8, 12 and 15 °C in Chinook salmon, Purcell et al. (2016) reported that mortalities, bacterial loads in the kidney as well as bacterial shedding rates were highest at lower temperature [30]. Moreover, Purcell et al. [30] have reported an increase in the numbers of outbreaks at low temperatures. Notably, water temperature influences the severity of the disease with low temperatures being associated with a more chronic presentation [24].

### 4.2. Susceptible Species

*R. salmoninarum* is often considered to have a limited host range. However, it is accepted that it can infect all members of the *Salmonidae* family (see Supplementary Table S1), including Chinook salmon, Coho salmon, rainbow trout [31,32,33]. Susceptibility varies between species: pink (*Oncorhynchus gorbuscha*), sockeye (*Ocorhynchus nerka*) and Chinook salmon (*Oncorhynchus tshawytscha*) are considered particularly susceptible to this disease in comparison with Atlantic salmon (*Salmo salar*) or *O. mykiss* [8,34].

In addition, while BKD is mostly known as a disease of salmonid fish, infection has also been reported from a few non-salmonid species, such as ayu (*Plecoglossus altivelis*). Artificial infection has also been performed in several more species, including Pacific herring (*Clupea harengus pallasi*), sablefish (*Anoplopoma fimbria*), shiner perch (*Cymatogaster aggregate*), common shiner (*Notropis cornutus*) and flathead minnow (*Pimephales promelas*) [24,35,36] as well as flathead (*Platycephalus indicus*) [37,38]. *R. salmoninarum* was identified by Chambers et al. [39] from eel in the river catchment in England using PCR in 2008 [39]. Minnow (*Phoxinus phoxinus*) and three-spined stickleback (*Gasterosteus aculeatus*) have also been reported to be sensitive to this disease [40].

The susceptibility of marine species to *R. salmoninarum* has not been commonly observed. However, this organism was detected in herring (*Clupea harengus*) by enzyme-linked immunosorbent assay (ELISA) [41]. *R. salmoninarum* antigens have also been identified in flathead (*Platycephalus indicus*) and Japanese sculpin (*Cottus japonicus*) by indirect dot blot assay and indirect fluorescent-antibody technique using polyclonal and monoclonal antibodies. Likewise, *R. salmoninarum* was isolated from flounder (*Limanda herzensteini*), greenling (*Hexagrammos otakii*), Japanese sculpin and flathead that had been captured from Coho salmon sea cages in Japan [38] as well as in sea lamprey (*Petromyzon Marinus*) in Lake Ontario using nested PCR and ELISA [42].

Blue mussel (*Mytilus edulis*) is able to filter *R. salmoninarum* out of seawater and to disrupt horizontal transmission and might play a role as a reservoir on the salmon farm [37]. Similarly, scallops (*Patinopecten yessoensis*) have been shown to carry the bacteria [38]. In conclusion, the list of species susceptible to being infected, or at least acting as a carrier for *R. salmoninarum*, is in fact larger and more diverse than is often assumed.

## 5. Routes of Infection and Risk Factors of *R. salmoninarum*

BKD can be transmitted directly without intermediate host via horizontal and vertical transmission. Horizontal routes of entry include the gastro-intestinal tract as well as through skin lesions and the eyes [43,44]. Important sources of infection are contaminated feed and water as well as cannibalism of infected fish or feeding salmon raw contaminated fish viscera [24,45,46]; however, this bacterium does not survive processing [26]. The feces of infected fish can constitute an important source of BKD in seawater net-pens [3].

Horizontal transmission via water is considered to be limited since *R. salmoninarum* is not able to survive outside the host for longer than a week [2,4,24]. Nonetheless, transmission has also been reported between cages in farm and farm to farm as well as from wild or escaped fish that play a role as a reservoir [2], a recent survey conducted in Austria showed that the bacterium was present in low numbers even in externally healthy wild fish populations [47]. Moreover, sea lice (*Lepeophteirus salmonis*) have been suspected to act as a vector; however, this has not been confirmed, even if treatment against sea lice is known to have a significant impact on the occurrence of BKD [4,20].

Another important feature of the disease is the existence of vertical transmission. The organism can be found in coelomic fluid [48] and enters the egg yolk during fertilization [49]; therefore, disinfection of the egg surface does not result in the elimination of the bacterium [43].

Several risk factors can contribute to the outbreak of BKD. These include low levels of dissolved oxygen, pollution, and the presence of harmful plankton as well as insufficient feeding [50,51]. In addition, stress during transfer or handling can act as contributing factors in the outbreak of disease or heighten mortality [4,51], as does stress due to changes in temperature [4].

## 6. Clinical Signs and Diagnosis

### 6.1. Clinical Signs

**Figure 1:**
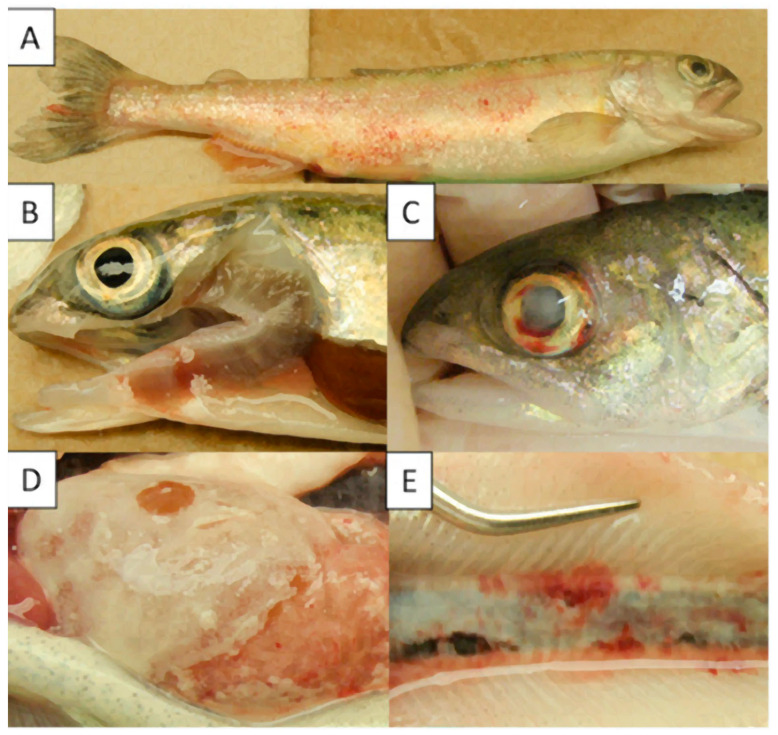
Clinical signs of bacterial kidney disease (BKD) in Chinook salmon, approximately 61–65 g and 19 cm in length and injected intraperitoneally with *R. salmoninarum* (2.1 × 10^10^ cfu/mL). (**A**): Haemorrhage and petechiae around lateral line; (**B**): pale gill; (**C**)): corneal opacity with associated haemorrhage; (**D**): false membrane covering the liver; (**E**): granulomatous lesions in the kidney [52].

BKD is a chronic condition and is typically associated with sustained occurrence of low levels of mortality, which can make detecting the presence of a problem difficult. Externally, fish infected with *R. salmoninarum* tend to lose equilibrium, and show irregular swimming behaviour. Additional external signs consist of unilateral or bilateral exophthalmia [20] alongside ocular lesions [52]. Blebs and blister on the epidermis with white or yellowish hemorrhagic fluid [9], haemorrhages or petechiae around the fins and lateral line [52], skin discoloration and dark pigmentation, swelling of the abdomen, pale gills (anaemia), anal vent haemorrhage as well as corneal opacity have also been reported. Moreover, adult infected fish display belly rash on the skin as well as muscles cavitation [24,36,43] (Figure 1A–C).

Internal signs consist of swelling of the kidney, heart, spleen and liver with creamy-white and greyish granulomatous lesions on the surface of the viscera with leucocyte, bacteria and host cell debris [9]. More occasionally, haemorrhage in the internal organs (hindgut and peritoneum) and muscle [20], bloody ascites fluid accumulation in the abdominal cavity and the covering organs with a false membrane have been reported, especially in fish kept at a low temperature, suggesting that this might be a more chronic presentation of the disease [9,18,53,54]. Moreover, brain lesions have been reported from several species (rainbow trout, brook trout, Atlantic, Chinook and Coho salmon) following the invasion of the central nervous system (CNS) via the posterior ocular urea [24,36] (Figure 1D,E).

### 6.2. Histopathology

BKD is characterized histologically by the development of chronic inflammation, granulomas and non-encapsulated granulomatous lesions, diffuse and with poorly defined borders throughout the viscera with caseation in the centers and proliferation of epithelioid macrophages (melanomacrophages) in the kidney, liver as well as the spleen alongside degeneration glomerulus and alteration of renal corpuscles and tubules. Moreover, glomerulopathy or glomerulonephritis has also been reported in the renal kidney. Additionally, uriniferous tubules and glomeruli are surrounded by hematopoietic tissues including macrophages, lymphocytes, neutrophils as well as eosinophils. Occasionally, adherence in the Bowman’s capsule has been reported in this disease [34]. In the spleen, *R. salmoninarum* enter the lymphohaemopoietic and splenic parenchyma causing focal tissue damage. The presence of monocyte-like cells and infected macrophages and barrier cells are reported [55].

The heart constitutes another target organ for this organism and infected fish can develop spongy and necrosis myocardium as well as granulomatous myocarditis consisting of eosinophil, karyorrhectic debris, macrophage and neutrophils [56,57]. Degeneration of myofibers (hypertrophied, pale and vacuolated), and diphtheritic epicarditis lesions in the heart tissue have also been reported [34,56,57].

Histopathological alterations in the liver include separation of hepatocytes and existence of fibrin within sinusoids [34,56,57]. Furthermore, Speare in 1997 reported that *R. salmoninarum* could be associated with neural pathology in the brain and described encephalitis and meningitis in Atlantic salmons and Chinook salmons [34,56,57,58].

### 6.3. Cultivation and Biochemical Profile

*R. salmoninarum* is a slow-growing fastidious pathogen, and specific media have been developed for this bacterium [38]; these include kidney disease medium-1 (KDM-1), KDM-2, KDM-C (with charcoal) as well as selective kidney disease medium (SKDM). When cultivated on KDM-2, the organism produces creamy, smooth and shiny colonies [59,60] and the bacterium is known to grow in Ten-M medium [61]. Similarly, it has been shown that this organism is able to grow in KDM-2 medium with culture-spent medium of *R. salmoninarum* (SMRs) instead of fetal bovine serum (FBS) [62].

Microaerophilic cultivation can increase the ability of *R. salmoninarum* to grow in KDM-2. Cultivation in microaerophilic condition may be used to increase the recovery of this organism, especially when present in low numbers [63]. *R. salmoninarum* is generally cultivated at temperatures ranging from 15 to 18 °C, forming visible colonies in 2 to 6 weeks. However, cultivation on KDM-2 might be time-consuming (up to 8 to 12 weeks) [64,65,66]. Isolation of the organism is therefore difficult and time consuming, and is not suitable as a diagnosis method.

Because of the slow-growing nature of *R. salmoninarum*, there is a risk for the medium to become contaminated with heterotrophic bacteria, which will overgrow *R. salmoninarum*. Antimicrobial agents (for example, 0.005% w/v cycloheximide, 0.00125% w/v D-cycloserine, 0.0025% polymyxin B sulfate or 0.00025% oxolinic acid) can be added to the medium to reduce the risks of such contamination [67], and enrichment with an antibiotic may reduce the risk of contamination with fungal or other non-specific bacterial agents [65].

The bacterium shows a positive reaction for catalase, alkaline phosphatase, acid phosphatase, esterase lipase, caprylate esterase, leucine phosphatase, leucine arylamidase, phosphomidase, α-glucosidase, α-mannosidase, casein hydrolysis, trypsin, Tween-20, 40 and 60 hydrolysis and also negative reaction for butyrate esterase, chymotrypsin, cystine arylamidase, α-fucosidase, α- and β-galactosidase, β-glucosaminidase, myristate esterase, oxidase, valine arylamidase, arginine hydrolysis, amylase and nitrate reduction [22,28,67].

### 6.4. Immunodiagnostic Methods

Several immunohistochemical techniques have been developed for the diagnostic of *R. salmoninarum* infections and these were once the method of choice for the detection of this pathogen. For example, Brattgjerd et al. (1994) has described the use of two antibodies raised against the membrane protein *p57* for immunohistochemistry [68]. Similarly, Bullock and Stucky [69] used fluorescent antibody technique (FAT) prepared against whole-cell antigens of *R. salmoninarum* to identify the presence of the pathogen and Elliott and Mckibben [70] have reported that Membrane-Filtration - Fluorescent Antibody Technique (MF-FAT) was more sensitive than Smear-FAT (S-FAT) to identify low numbers of *R. salmoninarum* [70]. Moreover, Dot-blot has also been used to detect the presence of *R. salmoninarum* using polyclonal antibodies against this bacterium [71]. ELISA procedures have been developed to detect heat shock protein 70 (HSP-70) [72]. Intriguingly, there is only a low correlation between the results of ELISA and FAT [73]. Similarly, Kent et al. [57] reported a lack of agreement between direct fluorescent antibody test (DFAT) and ELISA [57]. Moreover, comparison studies have shown that polyclonal ELISA is more sensitive compared to monoclonal ELISA [74,75].

While molecular methods have largely replaced immunodiagnostic as the primary method for the diagnostic of BKD, antibody-based methods still have a place in the control of the disease; in some cases, in particular in fish recovering from the disease, it can be more sensitive than molecular methods. Moreover, serum sampling is a non-lethal method, which makes it suitable for the routine screening of populations, notably of broodstocks.

### 6.5. Molecular Diagnostic

**Figure 2:**
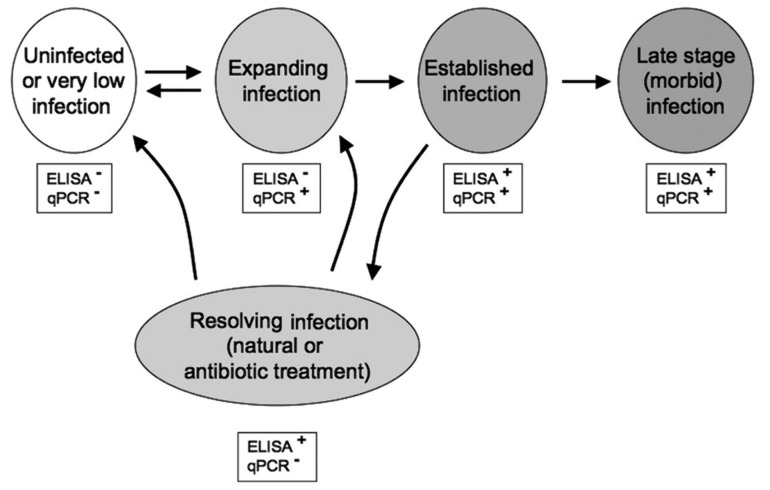
Correlation between ELISA and quantitative PCR (qPCR) assays for the identification *R. salmoninarum* in various types of infection [77]© Inter-Research 2010.

Compared to other techniques, when performed accurately, molecular diagnosis is more sensitive and therefore particularly useful for fish with subclinical infection [16,75,76] However, in some circumstances, for example, when the bacterial levels were low following antibiotic treatment (erythromycin or azithromycin), ELISA could detect infection while qPCR could not (Figure 2) [77].

Several PCR primers have been developed to detect the msa gene, 16S rRNA, p57 gene and ELF (elongation factor alpha 1) of *R. salmoninarum* [78,79,80,81].

Among the other molecular methods that have been developed are nested polymerase chain reactions (nPCR) [82], as well as real-time quantitative PCR (qPCR) [81,83,84]. The Office International des Epizooties (OIE) has recommended several preferred diagnostic procedures, including a PCR method developed by Brown et al. [85] for the identification of the *R. salmoninarum p57* gene. The authors indicated that this method is useful for detecting *R. salmoninarum* in very low doses in eggs [85]. Moreover, Cook and Lynch in 1999 suggested a nested reverse transcriptase PCR (RT PCR) for the identification *R. salmoninarum* based on mRNA in kidney tissue and ovarian fluid from Atlantic salmon [82]. Similarly, PCR assays have been performed to detect *R. salmoninarum* in rainbow trout head kidney lymphocytes and could detect this pathogen after 4 days post infection even at low level of bacteria (6×10^2^ CFU/mL) [86]. However, no bacteria were observed in kidney samples on the day of the infection, suggesting that it takes some time for this organ to accumulate a detectable level of bacteria [86].

It has been reported that qPCR had a higher sensitivity and specificity compared to other molecular methods [87]. Comparison between three qPCR assays including *msa*/non-fluorescent quencher (*msa*/NFQ), abc/NFQ assay and PCR using abc primers and a TAMRA quencher for the identification of *R. salmoninarum* in Chinook salmon and Coho salmon has suggested that *msa*/NFQ and abc/NFQ assays were more sensitive [88]. Recently, González-Rentería et al. (2019) reported that the sensitivity and specificity of nPCR could be improved by using different temperatures and cycling primers’ alignment to avoid a non-specific banding pattern on gel electrophoresis and reported a detection limit of 6.5 cells of *R. salmoninarum* [89].

More recently, loop-mediated isothermal amplification (LAMP) has been developed to detect *R. salmoninarum.* This approach has the advantage of sensitivity and specificity comparable to a PCR without the need for specific lab equipment and training, which could make it easier to apply in the field [66,90].

## 7. Virulence Mechanisms

Bacterial adhesion to the host cells is performed through the interaction of microbial surface components (MSCRAMs) such as the protein sortase with adhesive matrix molecules. Because phenyl vinyl sulfone (PVS) is able to inhibit sortase and reduce the adherence of *R. salmoninarum* to fish host cells, this drug can decrease the likelihood of a BKD outbreak in salmonid fish [91]. Following adhesion, an important feature of the disease is the ability of the bacterium to invade host cells and survive intracellularly [8], and notably, the ability to survive within macrophages [92]. This ability has been linked to the low efficacy of bacterial killed vaccines, as these would not stimulate the MHC I immune pathway [93,94].

*R. salmoninarum* infections have also been associated with changes in haematological and serum parameters, such as alteration in the haematocrit as well as a decrease in erythrocytes numbers and diameter. Other haematological cells, including neutrophils, monocytes and thrombocytes, have been shown to be more numerous following BKD, while lymphocyte rates remain unvaried during infection. Infection can also reduce cholesterol and sodium in the blood while increasing the serum bilirubin, potassium and blood urea nitrogen [8,34].

The best-characterized virulence factor of *R. salmoninarum* is *p*57, a 57-kDa protein present on the bacterial cell surface as well as secreted into the surrounding tissue. *P57* is encoded on three copies of *msa* (*msa1* and *msa2*) and *msa3. Msa1* and *msa2* have identical nucleotide sequences, save for a mutation in the amino-terminal region, where Ala139 is replaced with Glu. Both *msa1* and *msa2* are necessary to the full virulence of *R. salmoninarum* [80], and it has been reported that the presence of a *msa3* encoding gene was correlated with virulence and mortality even at a low number of bacterial pathogens [95,96,97]. The secretion of *p*57 and *p22* is known to agglutinate salmon leukocytes. Moreover, *p57* as well as *p22* have immunosuppressive properties, suppressing the production of specific antibodies in salmonid B cells and both of them have an effect on virulence factor [98].

Analysis of the genome of the ATCC 33209^T^ strain revealed multiple genes involved in iron metabolism, including ABC transporters, haemin transport systems (*Hmu*), major facilitator superfamily enterobactin (siderophore)-related transport, and IdeR/DtxR regulator, as receptors, in siderophore biosynthesis and enterobactin esterase, that play a role in expressing protein for iron uptake [99,100]. Grayson et al. [100] investigated iron acquisition in *R. salmoninarum* and have reported that non-hydrophobic strains were not able to grow properly under iron-restricted conditions compared with hydrophobic strains. Additionally, two iron acquisition systems (siderophores and haeme) have been described by Bethke et al. [101]. Recently, Bethke et al. [101] investigated the expression of bacterial genes and host immune genes during in vitro infection of a cell line (Atlantic salmon kidney cells) and culture under iron-limited conditions. These authors reported that several bacterial genes such as *HmuTUV* cluster, the siderophore transporters FecB and FecD, the siderophore esterase Est.1 and Est.2 and the ferrous transporter FTR-1 were overexpressed under iron limited conditions, suggesting that these genes play a role in iron acquisition. While this study was performed in vitro, these findings might be useful for creating novel treatment via disrupting the iron uptake [101].

Additionally, extra-cellular proteins of *R. salmoninarum* have also demonstrated limited proteolytic activity [102]. The presence of two haemolytic enzymes (metalloprotease and haemolysin), one encoded by the gene *rsh* and the other, a metalloprotease, encoded by the *hly* gene, has also been demonstrated [103]. Another putative virulence gene (*glcK*) encoding glucose kinase has also been found to be over-expressed during infection [104]. Comparison of the genome of two Chilean isolates (H2 and DJ2R) with that of the ATCC 33209^T^ strain indicated that the Chilean isolates possessed additional metabolic pathways such as the tricarboxylic acid cycle, glycolysis pathway and possessed additional iron transporters as well as revealed some proteins homologous with protein from *Mycobacterium* spp. [105].

Moreover, the immuno-suppressive ability of *R. salmoninarum* means that other pathogens such as Gram-negative *Aeromonaceae* can take advantage and cause secondary infections [106]. Despite these findings, it is clear that our understanding of the virulence mechanisms of *R. salmoninarum* remains incomplete and most of the research so far has focused on *p57.* While other potential virulence factors have been reported, our understanding of their mechanisms of action is still very limited.

## 8. Treatment, Control and Disinfection Equipment of *R. salmoninarum*

BKD is one of the most difficult fish diseases to control. No efficacious vaccines are currently available and it is known that wild fish can act as a reservoir and vector for the disease [8] and no therapeutic procedures are available to completely eliminate *R. salmoninarum* in salmonid fish population [64].

### 8.1. Selection

Prevalence of diseases can be decreased through selective breeding programs. When genome wide-analysis was applied using a 50K single nucleotide polymorphism (SNP) panel to investigate intra specific differences of sensitivity in 652 post-smolts originating from 63 families, two markers were found associated with resistance [107]. The genome-wide association has been measured for binary survival for 44527 SNP markers. The first SNP marker, Ssa04, was significantly associated with the time required for the bacteria to lead to a fatal outcome, while the second marker, Ssa08, was associated with survival. However, the correlation of these markers with resistance was limited, 5.3% and 4.0%, respectively. In addition, some SNP markers have been detected in this study, such as 10 significant SNPs in Ssa04 chromosomes, 4 SNPs in *Ssa*05 as well as 6 SNPs in *Ssa25* [105]. Similarly, genetic variation in the resistance to BKD has been described in Atlantic salmon with European region by Gjedrem and Gjøen in 1995. They also found correlation between survival and genetic variation [108]. These results suggest that improved resistance against *R. salmoninarum* could be achieved through breeding programs.

### 8.2. Vaccination

Classically, vaccine development has focused on the use of whole-cell inactivated bacteria, mostly using heat or formalin to inactivate the pathogen, while delivery has generally been performed by injection, intraperitoneally or, less commonly, intramuscularly, often alongside an adjuvant such as Freund’s incomplete adjuvant (FIA). However, when compared to delivery through the feed, it was found that oral delivery could result in an improved immune response in Coho salmon [93]. Over time, several attempts have been made at developing bacterin-killed vaccines against BKD, for example, by heat inactivation of the bacterium. Formalin-killed bacterin have been shown to stimulate the expression of immune and immunoregulatory genes, including *tlr5* and *clec12b* as well as *tnfrsf6b* and *tnfrsf11b* [109]. Similarly, specific immunogenic surface proteins have been identified in *R. salmoninarum* such as the major soluble antigen (MSA) [59], suggesting that it should be possible to develop a vaccine against this pathogen. Nonetheless, these attempts have not resulted in the development of reliable protection, a fact that has been attributed to the facultative intracellular lifestyle of the bacterium, as killed bacterin vaccine do not stimulate the MHC I pathway [93,94].

Correspondingly, subsequent efforts have focused on the development of attenuated vaccinal strains. For example, Daly et al. [110] have reported on the use of two attenuated nutritional mutants (Rs TSA1 and Rs BHI) alongside Rs MT-239, an attenuated strain with reduced expression of *p57* on the bacterial cell surface. This experiment was performed in Atlantic salmon and the efficacy of these live vaccines was evaluated with killed vaccines containing complete Freund’s adjuvant. The results show varied levels of protection as mortality reached 100% in the group vaccinated with the killed vaccine. Meanwhile, mortality was only approximately 20% in the group that had received 5×10^6^ live cells of Rs TSA1 after 60 days post infection [110], corresponding to a relative percent survival value (RPS) of 80%.

Another source of potential live vaccines for *R. salmoninarum* is its avirulent environmental relatives belonging to the genus *Arthrobacter.* For example, *Arthrobacter davidanieli* has been shown to be able to elicit good protection against BKD in Atlantic salmon with survival of up to 80% [111,112]. This has led to the development of the commercial vaccine Renogen^®^ that is based on a live *Arthrobacter* species and is generally delivered by intraperitoneal injection. Renogen^®^ has been associated with high survival compared with non-vaccinated fish in field trials in Atlantic salmon [113,114]. However, this vaccine is only commercially available in the USA, Canada and Chile [34,113,115]. Conversely, comparison between multiple vaccine solutions, including the attenuated and killed MT-239, the killed Rs 33209, killed Rs 33209 (heated for 48 h at 37 °C), Renogen^®^, PBS and PBS emulsified with FIA, showed no significant differences between the protection levels of these vaccines and phosphate-buffered saline (PBS) or PBS emulsified with FIA [116]. Additionally, the efficacy of a whole bacterial cell vaccine alongside a genetic adjuvant was evaluated in juvenile Chinook salmon by Rhodes et al. in 2004 [117] and it was found that a combination of Renogen^®^ and killed MT-239 resulted in increased survival (more than 60% survival rate after 98 days initial vaccination) following challenge by intraperitoneal injection [117]. These results suggest that the immune response induced by these vaccines might vary from one vaccination to the other and might not be reliable.

### 8.3. Antibiotherapy

The first antibiotic therapy against *R. salmoninarum* was performed by Rucker et al. in 1951 in Blueback salmon (*Oncorhynchus nerka*) using sulfadiazine (264 mg/kg) for 7 days followed by a reduced dose of 132 mg/kg for the following 21 days [118]. Since then, multiple other antibiotics have been applied in the treatment of BKD, including erythromycin (100 mg/kg for 21-28 days) [119,120], azithromycin (30mg/kg for 14 days) [120], as well as enrofloxacin, commercialized by the Bayer company under the name “Baytril^®^“ [22,121]. Notably, the use of erythromycin in fish is not allowed in the United States as therapeutic drug. It is only accessible as INAD (investigational new animal drug) accorded by the food and drug administration [8,122].

Because of the bacterium’s limited susceptibility, antibiotic therapy requires prolonged treatment—up to 28 or 30 days [123]. Additionally, due to the intracellular location of *R. salmoninarum*, antibiotic therapy is not very effective [43]. More importantly, instances of antibiotic resistance have been reported [8,124]. For example, screening of seven strains of *R. salmoninarum* showed decrease susceptibility to macrolide antibiotics. Different classes of macrolide resistance genes have been identified on the genome of *R. salmoninarum*, including genes encoding two 23S rRNA methyltransferases (*rlmA* and *sopU*), macrolide efflux factor (*mefA*), multidrug resistance efflux pump (*pvsC*) and *ksgA* (16S rRNA dimethylase) [25,123,125].

Antibiotics remain the most commonly applied method for the control of outbreaks of bacterial diseases and this overreliance has several implications, both in term of environmental and public health. In this context, it is vitally important to reduce the application of antibiotics, and, in particular, to find reliable alternatives to these overused treatments.

### 8.4. Nutritional Supplements

The observation that the levels of several minerals and vitamins (vitamin A, zinc and iron) were lower in the kidney and liver of infected fish prompted researchers to investigate the benefit of feeding compensating diets to the fish. Correspondingly, it was shown that feeding supplements with high levels of elements such as copper, iodine, iron, manganese and fluorine could result in a decreased number of bacteria [126]. Similarly, dietary supplementation using zinc, iron, manganese and sodium-L-ascorbate was shown to affect survival time when the food has low levels of zinc and manganese [127]. Conversely, the ability of vitamins A, E, D, B1, B2, B6, C and K and the minerals calcium, folic acid and inositol to reduce mortality in Masu salmon in a hatchery was also investigated but the results indicated no effect on the mortality rate. Moreover, Thorarinsson et al. [128] revealed that vitamin E and selenium did not influence the prevalence of *R. salmoninarum* in Chinook salmon [128,129]. Furthermore, dietary composition such as iodine and fluorine was shown to effect the likelihood of a BKD outbreak [26].

### 8.5. Culling and Segregation Infected Brood Stock

Because neither vaccination nor treatments are reliable for the control of the disease, the culling of infected organisms, in particular, culling gametes from infected animals, is still commonly practiced [5,64,130]. Because it allows for reducing the total number of animals unnecessarily sacrificed, this approach greatly benefits from the use of non-lethal sampling techniques such as ELISA and MF-FAT that permit to detect *R. salmoninarum* in non-lethal samples such as fin clips, gill snips, surface mucus scrapings, blood draws, or kidney biopsies and uro-fecal samples. Moreover, the sensitivity of monitoring programs can be increased by using a combination of lethal and uro-fecal sampling [52,131]. A pilot study performed in Iceland resulted in the prevalence of positive brood fish being reduced from 35% to less than 2% over 6 years of systemic culling of infected broodstock. Due to the sensitivity and specificity of ELISA for the detection of *R. salmoninarum* in broodfish population, this method can play a significant role in culling programs [132] as can the combination of methods such as RT-rPCR and ELISA [133]. However, because of the risk of reservoir populations in the wild, elimination of *R. salmoninarum* is not feasible via the culling method alone [8,134]. In our opinion, the culling method is a good approach to reduce the prevalence of the disease however; it is not sufficient alone and needs to be applied alongside other approaches such as vaccination and quarantine. Moreover, it is probably not reliable when a large population of wild susceptible fish are present, as these could likely act as reservoir for the disease.

## 9. Conclusions

BKD caused by *R. salmoninarum* infections can have serious enduring adverse effects on salmonid fish and is associated with increased morbidity and mortality in fish farms. Because of its subclinical form and vertical transmission, the bacterium is difficult to screen and control. Currently, our understanding of the mechanisms of virulence of *R. salmoninarum* is still very limited and further studies are required to better our understanding. This is all the more true because *R. salmoninarum* is such a unique pathogen as its closest relatives are considered avirulent environmental bacteria. A better understanding of the virulence of *R. salmoninarum* could give us insights regarding the acquisition of virulence in Gram-positive pathogenic bacteria. Similarly, a better understanding regarding the immune defense response against *R. salmoninarum* infection in fish would contribute to developing better prophylaxis and vaccine responses. Efforts regarding the development of live attenuated vaccinal strains need to be continued.

## Supplementary Materials

The following are available online at https://www.mdpi.com/2076-0817/9/10/845/s1, Table S1: Geographical distribution for *R. salmoninarum*.

## List of Abbreviations

Bacterial kidney disease (BKD); 16S ribosomal RNA (16S rRNA); Single-nucleotide polymorphism (SNP); Copy number variation (CNV); Whole genome sequencing (WGS); chrome azurol S (CAS); Interleukin-1β (IL-1β); Major histocompatibility complex class II (MHC II); Cyclo-oxygenase-2 (Cox-2); Inducible nitric oxide synthase (iNOS); Tumour necrosis factor-a (TNF-α); Central nervous system (CNS); Kidney disease medium (KDM); Selective kidney disease medium (SKDM); Culture-spent medium of *R. salmoninarum* (SMRs); Fetal bovine serum (FBS); Fluorescent antibody technique (FAT); Membrane filtration-FAT (MF-FAT); smear-FAT (S-FAT); Enzyme-linked immunosorbent assay (ELISA); Heat shock protein 70 (HSP-70); Polyclonal enzyme-linked immunosorbent assay (pELISA); Elongation factor alpha 1 (ELF); Nested polymerase chain reactions (nPCR); Quantitative PCR (qPCR); *major soluble antigen*/non-fluorescent quencher (*msa*/NFQ); Terminal restriction fragment length polymorphism (T-RFLP); Loop-mediated isothermal amplification (LAMP); Real- time reverse transcriptase PCR (RT-rPCR); Phenyl vinyl sulfone (PVS); Major soluble antigen (MSA); Single nucleotide polymorphism (SNP); Phosphate buffered saline (PBS); Minimum inhibitory concentration (MIC); Food and drug administration (FDA); Macrolide efflux factor (*mefA*); Multidrug resistance efflux pump (*pvsC*).
