# Supplementary material for: Renibacterium salmoninarum—The Causative Agent of Bacterial Kidney Disease in Salmonid Fish
Source: Pathogens. 2020 Oct 15;9(10):845. doi: 10.3390/pathogens9100845 (PMC7602803; doi:10.3390/pathogens9100845)
Supplement: Supplementary file 1 [file pathogens-09-00845-s001.pdf]

Supplementary Table 1: Geographical distribution for *R. salmoninarum*

Place of isolation	Affected species	Reference
Pacific Ocean	Chinook trout (*Oncorhynchus tshawytscha)*, coho salmon (*O. Kisutch),* chum salmon (*O. Keta),* pink salmon (*O. Gorbuscha),* socheye salmon (*O. Nerka), Salmo gairdneri, S. clarkia*	[1,2]
Alaska	Anadromous Pacific salmon (*Oncorhynchus spp.*)*,* non-anadromous Dolly Varden (*Salvelinus malma*), Arctic char (*S. alpinus*), Arctic grayling (*Thymallus arcticus*), brook trout (*S. fontinalis*), cutthroat trout (*O. clarki*), lake trout (*S. namaycush*), rainbow trout (*O. mykiss*), steelhead (anadromous rainbow trout)	[3]
Ontario (Canada)	Rainbow trout (*O.mykiss*)*,* lake trout (*Sal velinus namaycush*)*,* brook trout (*S. fontinalis*)*,* brown trout (*Salmo trutta*), Atlantic salmon (*Salmo salar*), coho salmon (*O. kisutch*), chinook salmon (*O. tshawytscha*), walleye (*Stizostedion vitreum*), and lake whitefish (*Coregonus clupeaformis*), Arctic char (*Salvelinus alpinus*)	[4]
Germany	*Hucho hucho, Salvelinus alpinus*	[5]
Michigan (Michigan river), Oregon (Columbia and Snake river), Washington and Wyoming (North America)	Chinook salmon (*O. tshawytscha*)*,* brook trout (*Salvelinus fontinalis*), brown trout (*Salmo trutta*), and rainbow trout (*Salmo gairdneri*)	[6–8]
Loon Lake (British Colombia)	Rainbow trout (*O. mykiss*)	[9]
Poland	Brook trout (*Salvelinus fontinalis*)	[5]

References:

1. Buller, N.B. *Bacteria and Fungi from Fish and other Aquatic Animals: a practical identification manual*; 2nd ed.; CABI Publishing: Wallingford, United-Kigdom, 2014; p. 904.
2. Banner, C.R.; Long, J.J.; Fryer, J.L.; Rohovec, J.S. Occurrence of salmonid fish infected with *Renibacterium salmoninarum* in the Pacific Ocean. *J. Fish Dis.* **1986**, *9*, 273–275.
3. Meyers, T.R.; Korn, D.; Glass, K.; Burton, T.; Short, S.; Lipson, K.; Starkey, N. Retrospective analysis of antigen prevalences of *Renibacterium salmoninarum* (Rs) detected by enzyme-linked immunosorbent assay in Alaskan Pacific salmon and trout from 1988 to 2000 and management of Rs in hatchery Chinook and coho salmon. *J. Aquat. Anim. Health.* **2003**, *15*, 101–110, doi:10.1577/H02-022.
4. Bruneau, Nathallie &Thorburn., M. Occurrence of aeromonas salmonicida, renibacterium salmoninarum, and infectious pancreatic necrosis virus in Ontario salmonid populations. *J. Aquat. Anim. Health.* **1999**, *11*, 350–357, doi:10.1577/1548-8667(1999)011<0350:OOASRS>2.0.CO;2.
5. Pfeil-Putzien, C.; Hoffmann, R.; Popp, W. Preliminary report on the occurrence of bacterial kidney disease in Germany. *Bull. Eur. Assoc. Fish Pathol.* **1985**, *5*, 30–31.
6. Holey, M.E.; Elliott, R.F.; Marcquenski, S. V; Hnath, J.G.; Smith, K.D. Chinook Salmon epizootics in Lake Michigan: Possible contributing factors and management implications. *J. Aquat. Anim. Health.* **1998**, *10*, 202–210.
7. Vanderkooi, S.P.; Maule, A.G. Prevalence of *Renibacterium salmoninarum* in juvenile spring Chinook salmon at Columbia and snake river hatcheries, 1993 – 1996. *J. Aquat. Anim. Health.* **1999**, *11*, 162–169.
8. Mitchum, D.L.; Sherman, L.E.; Baxter, G.T. Bacterial kidney disease in feral populations of brook trout (*Salvelinus fontinalis*), brown trout (*Salmo trutta*), and rainbow trout (*Salmo gairdneri*). *J. Fish. Board Canada.* **1979**, *36*, 1370–1376.
9. Evelyn, T.P.T.; Hoskins, G.E.; Bell, G.R. First record of bacterial kidney disease in an apparently wild salmonid in British Columbia. *J. Fish. Board Canada.* **1973**, *30*, 1578–1580.
